# 免疫缺陷人群难治性/耐药性巨细胞病毒感染诊治策略中国专家共识（2025年版）

**DOI:** 10.3760/cma.j.cn121090-20250112-00024

**Published:** 2025-05

**Authors:** 中华医学会血液学分会感染学组 中华医学会器官移植分会 中国临床肿瘤学会白血病专家委员会 广东省医学会呼吸病学分会 广东省基层医药学会免疫缺陷专委会

## Abstract

我国免疫缺陷人群数量迅速增多，巨细胞病毒（cytomegalovirus，CMV）感染的发生率远高于正常人群，严重影响生活质量及预后。目前尚缺乏针对免疫缺陷人群难治性/耐药性CMV感染的标准化诊疗体系。本共识基于国内外难治性/耐药性CMV感染的流行病学、循证医学证据和临床研究等，针对难治性/耐药性CMV感染的诊断、治疗及管理形成了推荐意见。

## Abstract (en)

The number of immunocompromised individuals in China is rapidly increasing, and the incidence of cytomegalovirus（CMV）infection in this population is significantly higher than in the general population, severely affecting their quality of life and prognosis. Currently, a standardized diagnostic and treatment system for refractory/resistant CMV infections in immunocompromised populations is lacking. Based on domestic and international data on the epidemiology, evidence-based medicine, and clinical research regarding refractory/resistant CMV infections, this consensus formulates recommendations for the diagnosis, treatment, and management of these infections.

免疫缺陷人群是原发性和继发性免疫功能受损人群的统称。前者由先天性免疫缺陷病引起，后者由获得性免疫缺陷病引起。获得性免疫缺陷病又分为感染性和非感染性两种，感染性因素包括细菌、真菌及病毒等；非感染性因素包括恶性肿瘤、实体器官移植（solid organ transplantation，SOT）、异基因造血干细胞移植（allogeneic hematopoietic stem cell transplantation，allo-HSCT）、因风湿免疫性疾病等接受长期和（或）大剂量糖皮质激素及免疫抑制剂等[1]。近年来，我国免疫缺陷人群数量迅速增多，巨细胞病毒（cytomegalovirus，CMV）感染的发生率远高于正常人群，严重影响生活质量及预后，难治性和耐药性CMV感染也逐渐增加，导致患者死亡率显著上升，亟需临床高度关注。

目前尚缺乏针对免疫缺陷人群的难治性/耐药性CMV感染的标准化诊疗体系，因此，由中华医学会血液学分会、中华医学会器官移植分会、中国临床肿瘤学会白血病专家委员会、广东省医学会呼吸病学分会、广东省基层医药学会免疫缺陷专委会牵头，共同组织国内多个领域（器官移植科、血液科、感染科、呼吸与危重症医学科、重症医学科及风湿免疫科）专家，检索国内外相关文献，撰写《免疫缺陷人群难治性/耐药性巨细胞病毒感染诊治策略中国专家共识（2025年版）》。此共识基于国内外难治性/耐药性CMV感染的流行病学、循证医学证据和临床研究等，采用2009版牛津大学循证医学证据分级与推荐意见强度分级方法（见表1），经专家多次研讨并达成一致意见，供临床参考借鉴。

**表1:** 2009版牛津大学循证医学证据分级与推荐意见强度分级标准

推荐强度	证据级别	描述
A	1a	随机对照试验（RCT）的系统评价
	1b	结果可信区间小的RCT
	1c	显示“全或无效应”的任何证据
B	2a	队列研究的系统评价
	2b	单个队列研究（包括低质量的RCT，如失访率>20％者）
	2c	基于患者结局的研究
	3a	病例对照研究的系统评价
	3b	单个病例对照研究
C	4	病例系列报告、低质量队列研究和低质量病例对照研究
D	5	专家意见（即无临床研究支持的仅依据基础研究或临床经验的推测）

一、免疫缺陷人群难治性/耐药性CMV感染及CMV病的定义、流行病学

1. 免疫缺陷人群难治性/耐药性CMV感染及CMV病的定义：难治性CMV感染指在至少2周的适当抗病毒治疗后，CMV载量仍升高或持续存在的情况［同一类型的血液样本，在同一实验室检测和（或）使用相同的商业检测方法］[2]。其中，升高是指CMV DNA水平与峰值病毒载量相比增加>1 log10；持续存在指CMV DNA水平增加或减少≤1 log10。难治性CMV病指在至少2周的适当剂量抗病毒治疗后，体征和症状恶化或进展为终末器官疾病（之前未被诊断为患有CMV终末器官疾病）或体征和症状没有改善[2]。部分CMV感染患者出现骨髓抑制或肾功能损伤等不良反应，继而无法耐受抗CMV治疗，这部分患者在临床中也可被纳入难治性CMV感染或疾病。

耐药性CMV感染的定义：在符合难治性CMV感染诊断的基础上，有特定的基因突变导致CMV对1种或多种治疗药物的敏感性下降。

2. 免疫缺陷人群难治性/耐药性CMV感染的流行病学：不同免疫缺陷人群难治性/耐药性CMV感染发生率有所差异。allo-HSCT患者难治性CMV感染发生率为24％～39％，耐药性CMV感染发生率为1.7％～14.5％[3]。SOT患者难治性/耐药性CMV感染发生率因移植器官及免疫抑制方案的不同而有所差异[4]。在预防用药的SOT患者中，耐药性CMV的发生率为5％～12％，肺移植受者与小肠和多脏器联合移植受者耐药性CMV感染的发生率较高，分别为18％和31％[4]。人类免疫缺陷病毒（human immunodeficiency virus，HIV）感染者/获得性免疫缺陷综合征（acquired immune deficiency syndrome，AIDS）患者耐药性CMV感染的发生率约为19.5％[5]。

二、免疫缺陷人群难治性/耐药性CMV感染的高危因素

免疫缺陷人群难治性/耐药性CMV感染的风险因素包括既往接受过抗CMV治疗、抗病毒药物剂量不足、抗病毒药物暴露时间延长、抗病毒治疗期间病毒持续活跃复制、使用高效免疫抑制剂及暴露于耐药屏障较低的抗病毒药物[3],[6]。

SOT患者中，CMV D^+^/R^-^（D：供者；R：受者）和肺移植受者发生耐药的风险最高[7]。在HSCT受者中，难治性/耐药性CMV感染风险与高危CMV血清状态、非同胞相合移植、复发性感染及移植物抗宿主病（graft-versus-host disease，GVHD）等相关[3]。HIV感染者/AIDS患者发生难治性/耐药性CMV感染可能与未启动抗逆转录病毒治疗（antiretroviral therapy，ART）或者ART失败、CD4^+^ T淋巴细胞计数<50/µl、既往合并其他机会性感染、高病毒载量的CMV血症，HIV RNA>1×10^6^拷贝/ml等有关[8]。风湿免疫性疾病患者高龄、淋巴细胞计数较低、低白蛋白血症、使用免疫抑制剂和高剂量糖皮质激素可能是发生难治性/耐药性CMV感染的危险因素[9]。

三、难治性/耐药性CMV感染的实验室诊断

实验室诊断方法包括血清学、定量PCR、pp65抗原检测、病毒培养和组织病理学检查[10]。其中，定量PCR因其高灵敏度和高通量成为检测CMV DNA的首选，但每例受者进行连续病毒学监测时应注意标本的一致性，与血浆相比，全血检测的病毒载量值更高[11]。pp65抗原检测是用单抗检测CMV复制早期白细胞中表达的CMV pp65抗原，常作为备选的实验室检测方法[12]。

耐药性CMV感染的诊断常采用表型和基因型耐药检测。表型耐药检测的金标准是病毒斑点减少试验，但受限于对人员、技术和设施的要求高且时效性差，难以满足临床快速检测的需求[13]–[14]。因此，基因型耐药检测已成为评估耐药性CMV的首选方法，其中Sanger测序是目前最常用的方法[15]，但可能无法识别低频率和少数突变体毒株。而二代测序发展迅速，具备更高的分辨率和灵敏度，使更早期的抗病毒耐药性检测成为可能[16]。

四、免疫缺陷人群难治性/耐药性CMV感染的预防

问题1：哪些免疫缺陷人群适合预防性用药？

推荐意见1：对于SOT受者，建议对CMV D^+^/R^-^和R^+^受者进行预防性治疗（推荐强度：A，证据等级：1a）。

推荐意见2：对于HSCT受者，建议对CMV IgG阳性受者进行预防性治疗（推荐强度：A，证据等级：1b）。

推荐意见3：对于风湿免疫性疾病患者，在使用大剂量免疫抑制剂的同时，应当筛查CMV核酸，若没有CMV感染的证据，无需启动预防性治疗（推荐强度：D，证据等级：5）。

推荐意见4：对于HIV感染者/AIDS患者，不建议使用抗CMV药物进行一级预防。预防发生CMV血症和CMV病的关键是尽早启动ART，重建受损的免疫功能（推荐强度：B，证据等级：2b）。

推荐意见说明：

对于SOT患者，予D^+^/R^-^和R^+^受者预防性治疗通常利大于弊[17]。一项队列研究对应用预防性治疗或抢先治疗的D^+^/R^-^ SOT受者进行比较，接受预防性治疗组较抢先治疗组CMV病的发生率更低（8.6％对33.3％，*P*＝0.01）且未出现CMV终末器官疾病（0对5.7％，*P*＝0.22）[18]。

一项随机对照试验纳入接受HSCT的CMV血清阳性受者，与安慰剂组相比，接受来特莫韦预防性治疗的患者在第24周时CMV再激活显著减少（37.5％对60％，*P*<0.001）[19]。

对于风湿免疫性疾病患者和HIV感染者/AIDS患者，使用抗CMV药物进行一级预防成本较高、有发生耐药的风险且获益并不明确，且抗CMV药物存在多种不良反应，因此，不应进行预防性治疗[20]–[21]。一项前瞻性研究表明，ART能够有效预防AIDS患者感染CMV[22]。

问题2：预防性用药如何选择药物及确定疗程？

推荐意见5：对于SOT受者，常选择缬更昔洛韦和更昔洛韦预防性治疗，预防时间一般为3～6个月（推荐强度：A，证据等级：1b）。

推荐意见6：对于HSCT受者，使用来特莫韦可用于CMV感染的一级预防，但现阶段仍需积累中国人群的数据（推荐强度B，证据等级2b）。

推荐意见说明：对于SOT患者，预防性治疗最常用的药物是更昔洛韦和缬更昔洛韦[23]。在一项随机对照研究中，CMV D^+^/R^-^的SOT患者接受缬更昔洛韦或更昔洛韦预防性治疗，给药持续100 d，两组患者第12个月时CMV载量低于定量水平的比例相当（48.5％对48.8％），且安全性相似[24]。

对于HSCT患者，一项国外单中心回顾性研究显示，使用来特莫韦进行一级预防可使难治性/耐药性CMV感染风险降低85％[25]。国际指南建议来特莫韦预防一般应用100 d[26]，然而还应考虑是否合并GVHD、是否为CMV-IgG阴性供者以及CMV特异性细胞毒性T淋巴细胞（CMV-CTL）水平，酌情调整预防治疗的疗程。

五、免疫缺陷人群CMV感染的抢先治疗（或一线治疗）

问题3：免疫缺陷人群如何建立规范化抢先治疗（或一线治疗）监测干预流程？

推荐意见7：对于SOT和HSCT受者，当CMV DNA或CMV pp65抗原检测达到抢先治疗阈值时，即使临床未出现发热等症状，也建议采取抢先治疗（推荐强度：B，证据等级：2a）。建议每个中心根据患者的风险类别和特定中心的数据确定定量病毒载量阈值（推荐强度：A，证据等级：1b）。

推荐意见8：对于风湿免疫性疾病患者，若CMV DNA达到阈值，同时参考免疫抑制状态，考虑是否启动抢先治疗（推荐强度：D，证据等级：5）。

推荐意见9：对于HIV感染者/AIDS患者合并严重机会性感染（包括隐球菌性脑膜炎、结核性脑膜炎）或长期应用糖皮质激素而延迟启动ART患者，发生高病毒载量CMV血症时（CMV DNA≥1×10^4^拷贝/ml），可考虑进行抗CMV治疗（推荐强度：C，证据等级：4）。

推荐意见说明：抢先治疗是为预防CMV病发生而实施的针对CMV血症的治疗，一旦检出CMV血症阳性，原则上需积极干预[27]。对于SOT和HSCT患者，考虑到检测标本和检测平台的差异，建议每个中心根据患者的风险类别和特定中心的数据确定定量病毒载量阈值[26],[28]，国际标准建议以IU/ml为单位[29]。我国大多数中心采用CMV DNA水平连续2次>500拷贝/ml或单次>1 000拷贝/ml作为启动抢先治疗的标准[29]。

严重的风湿免疫性疾病患者往往需要大剂量免疫抑制剂治疗，应当积极筛查CMV感染。临床中，风湿免疫性疾病患者启动抢先治疗的时机可在血浆CMV DNA>1 000 IU/ml或全血CMV DNA>10 000 IU/ml的基础上，结合免疫抑制状态，考虑是否抢先治疗[30]。

对于HIV感染者/AIDS患者，当及时开始ART时，不建议进行抢先治疗[31]，一项随机对照试验结果表明，在开始ART的AIDS患者中，缬更昔洛韦组较安慰剂组并未减少CMV终末器官疾病的发生（4例对6例）[32]。

问题4：抢先治疗（或一线治疗）如何进行药物选择？

推荐意见10：对于免疫缺陷人群，抢先治疗的常用药物为缬更昔洛韦或更昔洛韦（推荐强度B，证据等级：2b）。

推荐意见11：对于因中性粒细胞减少不适宜口服缬更昔洛韦或静脉滴注更昔洛韦的患者，可考虑使用膦甲酸钠或马立巴韦进行抢先治疗（推荐强度A，证据等级：1a）；对于存在肾功能损伤且伴有中性粒细胞减少，不适宜使用膦甲酸钠的患者，可考虑应用马立巴韦进行抢先治疗（推荐强度A，证据等级：1a）。

推荐意见说明：在SOT患者中，一项随机对照试验发现缬更昔洛韦和更昔洛韦用于抢先治疗的成功率分别为82％和91％，且均无患者因不良事件退出研究[33]。

对于HSCT患者，一项回顾性研究比较了缬更昔洛韦和更昔洛韦在抢先治疗中的疗效及安全性，结果显示，两组患者CMV DNA载量有良好反应的比例分别为80.0％和75.7％，且未观察到严重不良反应和CMV相关疾病[34]。一项关于HSCT患者抢先治疗的多中心、双盲、随机、Ⅲ期试验结果显示，马立巴韦组约70％的患者在第8周时达到病毒血症清除，且中性粒细胞减少症发生率远低于缬更昔洛韦组（16.1％对52.9％），说明马立巴韦在抢先治疗中显示出更低的治疗限制性毒性[35]。

临床实践中，风湿免疫性疾病患者抢先治疗的方案一般参考移植领域，可以选择口服缬更昔洛韦或静脉滴注更昔洛韦。两种方案更昔洛韦血药浓度相近[36]，治疗时，可优先选择口服缬更昔洛韦。

对于HIV感染者/AIDS患者，可选用缬更昔洛韦、更昔洛韦或膦甲酸钠单药抢先治疗[21]。一项研究表明，使用更昔洛韦能够有效预防HIV感染者/AIDS患者发生CMV病并降低死亡率[37]。

六、免疫缺陷人群难治性/耐药性CMV感染/CMV病的治疗

问题5：如何管理难治性CMV感染或CMV病？

推荐意见12：对于免疫缺陷人群，应针对患者的个体化情况，谨慎减少免疫抑制剂的剂量，评估患者免疫状态，在此基础上考虑使用抗难治性CMV感染的药物治疗（推荐强度B，证据等级：2c）。

推荐意见13：难治性CMV感染或CMV病首选马立巴韦治疗（推荐强度A，证据等级：1a）。

推荐意见14：难治性CMV感染或CMV病也可考虑使用CMV-CTL进行过继性免疫治疗（推荐强度B，证据等级：2b）。

推荐意见15：对于移植后难治性/耐药性CMV再激活的管理，CMV特异性CD8^+^T细胞或可作为潜在的监测指标（推荐强度B，证据等级：2b）。

推荐意见说明：难治性/耐药性CMV的危险因素包括高效免疫抑制剂的使用，一项研究表明，减少免疫抑制剂的剂量似乎对早期CMV DNA血症清除有一定效果，但对CMV的根除率或复发率几乎没有影响[38]。

一项关于马立巴韦用于SOT和HSCT人群难治性/耐药性CMV感染或疾病的随机、开放、多中心、对照、Ⅲ期试验中，与研究者指定治疗组相比，马立巴韦组在第8周时CMV血症清除率显著升高（55.7％对23.9％，*P*<0.001），并维持至第20周；同时，马立巴韦组因治疗相关不良反应而停止治疗的患者更少（4.7％对23.3％）[39]。马立巴韦在难治性CMV感染或疾病的治疗中显示出更好的疗效，且治疗相关不良反应更少，是中国唯一获批用于成人移植后难治性/耐药性CMV感染或疾病的药物。

此外，复发性CMV感染与CD8^+^T细胞免疫重建不良相关[40]。难治性CMV感染或CMV病也可考虑使用CMV-CTL。但对于allo-HSCT患者，需排除活动性GVHD方可使用CMV-CTL进行过继性免疫治疗。我国不同中心开展了多个CMV-CTL治疗难治性CMV感染的研究，总体病毒清除率为67.5％～89.7％[41]–[45]，并发现供者来源与第三方来源的CMV-CTL疗效相似[46]。

一项研究对血清CMV阳性HSCT患者CMV特异性T细胞与CMV再激活的动力学进行回顾性分析，结果表明，CMV特异性CD8^+^T细胞计数与抑制CMV再激活具有强相关性。当CMV特异性CD8^+^T细胞持续低于阈值（925个/1 000 000个外周血单个核细胞）时，难治性/耐药性CMV会发生再激活，甚至发生CMV疾病[47]。

问题6：如何管理耐药性CMV感染或CMV病？

推荐意见16：当临床上怀疑CMV疾病患者出现抗病毒耐药时，应通过实验室检测确认，通常使用表型和基因型耐药检测（推荐强度A，证据等级：1b）。

推荐意见17：CMV感染一线治疗药物耐药后，可首选马立巴韦进行治疗（推荐强度A，证据等级：1a）。

推荐意见18：耐药性CMV感染或CMV病，可根据基因型耐药检测结果选择膦甲酸钠、西多福韦等药物（推荐强度B，证据等级：2c）。

推荐意见19：CMV-CTL也可考虑用于耐药性CMV感染或CMV病（推荐强度C，证据等级：4）。

推荐意见说明：当临床上怀疑CMV患者出现抗病毒耐药时，应通过耐药性检测确认，通常使用表型和基因型耐药检测。对CMV感染一线治疗药物耐药后，可首选马立巴韦进行治疗。

耐药性CMV感染或CMV病的治疗也可根据基因型耐药检测结果选择膦甲酸钠、西多福韦等药物[48]，常用方案见表2。然而，有病例报道及回顾性研究显示，使用大剂量更昔洛韦或膦甲酸钠治疗耐药性CMV疾病存在较强的骨髓抑制及肾毒性[49]–[51]。此外，仅少数病例报告显示来氟米特和青蒿琥酯可治疗难治性/耐药性CMV感染[52]–[53]，其临床效果仍存在争议。

**表2:** 不同基因型耐药性巨细胞病毒感染的药物选择

耐药基因型	药物选择
UL97突变型，更昔洛韦高水平耐药	① 转换使用马立巴韦； ② 转换使用膦甲酸钠； ③ 转换使用西多福韦
UL97突变型，更昔洛韦低水平耐药（M460I、C592G、L595W）	① 无巨细胞病毒病情况下，如耐受，可加大更昔洛韦剂量； ② 转换使用马立巴韦； ③ 转换使用膦甲酸钠； ④ 转换使用西多福韦
UL54突变型，更昔洛韦和膦甲酸钠耐药（±UL97突变）	① 转换使用马立巴韦； ② 转换使用西多福韦； ③ 可考虑增加辅助药物，如来氟米特或青蒿琥酯
UL54突变型，更昔洛韦和西多福韦耐药	① 转换使用马立巴韦； ② 转换使用膦甲酸钠； ③ 可考虑添加辅助药物，如来氟米特或青蒿琥酯
UL54突变型，仅膦甲酸钠耐药	① 转换使用马立巴韦； ② 转换使用更昔洛韦； ③ 可考虑增加辅助药物，如来氟米特或青蒿琥酯
UL54突变型，更昔洛韦、膦甲酸钠和西多福韦耐药	① 如耐受，继续使用膦甲酸钠并加用大剂量更昔洛韦； ② 转换使用马立巴韦； ③ 可考虑增加辅助药物，如来氟米特或青蒿琥酯
UL56、UL89、UL51突变型，来特莫韦耐药	转换使用马立巴韦、更昔洛韦、膦甲酸钠或西多福韦

使用抗CMV药物时，还需注意药物与常用免疫抑制剂、HIV感染常用药物及抗菌药物等的相互作用。治疗期间，应监测免疫抑制剂药物水平，根据需要调整免疫抑制剂的剂量。

输注CMV-CTL是治疗耐药性CMV感染或CMV病很有潜力的方法，已有多项病例报告报道了CMV-CTL成功治疗耐药性CMV感染或CMV病[54]–[55]。

七、展望

综上，对于免疫缺陷人群难治性/耐药性CMV感染的诊断、治疗和随访需要器官移植科、血液科、风湿免疫科、呼吸与危重症科、感染科、重症医学科等多学科联合协作。目前对于免疫缺陷人群难治性/耐药性CMV感染的诊治仍存在局限性：①研究或临床试验证据少；②缺乏适用于非移植、免疫功能低下患者的治疗指南；③尚需探索如何通过早期诊断和管理降低发病率和死亡率等，随着临床研究的深入，将对共识进一步完善，以推动我国免疫缺陷人群难治性/耐药性CMV感染防治事业的进步与发展。
